# Fortified food supplementation in children with reduced dietary energy and micronutrients intake in Southern Mexico

**DOI:** 10.1186/s12937-018-0385-3

**Published:** 2018-08-13

**Authors:** Gabriela Añorve-Valdez, Amado David Quezada-Sánchez, Fabiola Mejía-Rodríguez, Armando García-Guerra, Lynnette Marie Neufeld

**Affiliations:** 1 Instituto Nacional de Salud Publica (INSP), Cuernavaca, Morelos Mexico; 2 Autonomous University of the State of Morelos, Cuernavaca, Morelos Mexico; 3 Global Alliance for Improved Nutrition, Geneva, Switzerland; 4 Center for Nutrition and Health Research, INSP, Avenida Universidad 655, Colonia Santa María Ahuacatitlán, Cuernavaca, Morelos Mexico; 5 Center for Evaluation and Surveys Research, National Institute of Public Health, INSP, Cuernavaca, Morelos Mexico

**Keywords:** Children, Developing country, Dietary intake, Fortified food, Micronutrient powder, Supplementation

## Abstract

**Background:**

Nutritional supplements are an important source of complementary food for young children, since they may either complement or substitute nutrients obtained from other food sources. Assessing how the introduction of different types of supplements modifies the consumption of other food sources may help in designing supplementation programs that aim to improve the nutrition of vulnerable populations.

The objetive is to quantify dietary energy and nutrient intake among children aged 6–12 months who received one of three nutritional supplements.

**Methods:**

A cluster-randomized trial was conducted from 2005 to 2007. Urban communities were randomly allocated to one of three intervention groups receiving one of the following: a milk-based fortified food, micronutrient powders, or syrup. Each supplement was fortified with equal amounts of micronutrients. Dietary intake was estimated using a food frequency questionnaire (FFQ) to reflect the average consumption over the month prior to the interview. Children between 6 and 12 months of age were recruited. Median regression was performed with adjusted standard errors for clustered data, and the linear predictors for the median included the study group, study stage and their interaction. Adjusted medians by study group and study stage were obtained as post-estimations.

**Results:**

No statistically significant differences between study groups were observed at baseline. After four months of supplementation, the children in the fortified food group had a smaller increase in median dietary energy (183.7 kcal, CI95%: 59.9, 307.5) and dietary protein (6.6 g, CI95%: 2.6, 10.6) intake from their home diet than those in the syrup group (*p* < 0.05). These differences remained significant after adjusting for group differences at baseline. Regarding covariate-adjusted median changes from baseline to follow-up at 10 months, the children in the fortified food group had a smaller median increase in dietary energy intake than those in the syrup group (698 vs 915 kcal), with a difference of 217.9 kcal (CI95%: 20.4, 415.4).

**Conclusion:**

Children in the fortified food group consumed less dietary energy, protein, and micronutrients than those in the micronutrient powder and syrup groups. It is possible that absolute nutrient intake may be overestimated by the FFQ, but this possibility does not compromise the ability to compare study groups. Given the observed differences in dietary energy consumption among the three supplemented groups, it can be concluded that supplementation with micronutrient powders is an adequate option for urban children who have met their minimum energy and protein requirements.

## Background

Micronutrient deficiencies in early childhood (6 to 24 months) influence several functional outcomes, including linear growth, health, and immune function [1–9]. According to the 2006 National Health and Nutrition Survey (ENSANUT, by its Spanish acronym), 27.5% and 3.2% of children in Mexico under the age of five were zinc and folate deficient, respectively [10]. The prevalence of anemia in the 2012 ENSANUT survey was 38% in children aged 12–23 months [11]. Additionally, ENSANUT reported a prevalence of 9.7% for overweight-obesity in children under five years [11], raising serious concern about the changing dietary patterns of children.

The *PROSPERA Programa de Inclusión Social* (*PROSPERA*; formerly known as the *Oportunidades* Human Development Program [PDHO] and previously named *Progresa*) has been distributing complementary fortified food (FF) as part of the program’s benefits for children aged 6 to 59 months since 1997. The FF supplement provides 100% of the daily micronutrients and 20% of the daily energy requirements of these children [12, 13] and is intended for daily consumption as a mush, colloquially referred to as papilla.

In an early evaluation, the original program had significant effects on child growth in rural and urban areas and on anemia in rural areas [12, 14]. In-depth ethnographic studies showed a high acceptance of the FF, but patterns of intra-household sharing resulted in young children consuming far less than the recommended amount [15, 16]. It is important to assess dietary intake to understand the patterns of supplement utilization and the extent to which their utilization results in modifications of dietary intake of home foods.

Dietary intake was assessed as part of a cluster-randomized trial comparing the impact of 3 different types of nutritional supplements for children on several nutritional outcomes. The objective of the analyses presented here was to quantify the dietary energy and nutrient intake of children aged 6–12 months who received one of three nutritional supplements.

## Methods

The dietary intake and supplement consumption data reported here were collected as part of a cluster-randomized efficacy trial specifically designed to identify the best supplement for use in *PROSPERA* based on the comparative impact of selected nutritional outcomes, acceptance, consumption, and cost. The details of this intervention have been reported elsewhere, and the main results of the study have been published and presented at international conferences [12, 15, 17, 18].

### Design and participants

**Fig. 1:**
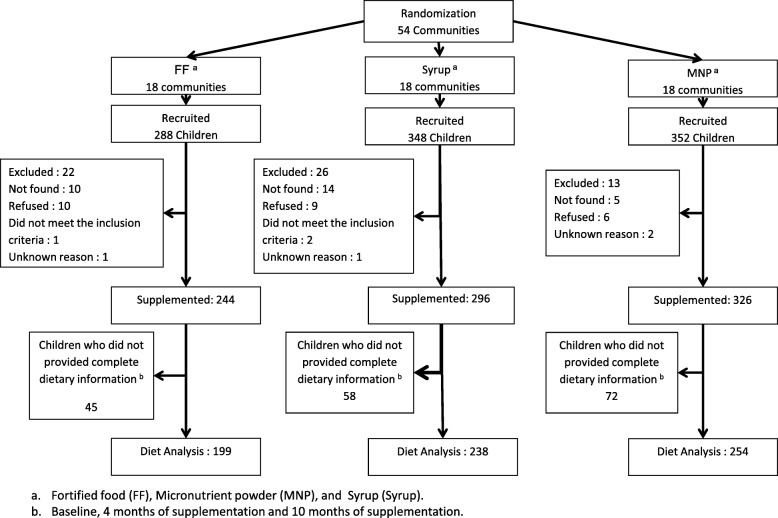
Flowchart of children participating in the study

We conducted a cluster-randomized trial from 2005 to 2007 in 54 urban communities in the Mexican states of Tabasco, Veracruz, Oaxaca and Puebla. Randomization was performed at the community level, and all *PROSPERA* beneficiaries who were children within the baseline age range in those communities were eligible to participate. Allocation was performed using a block design to ensure comparability between supplementation groups. The communities were split into 18 blocks of three communities each prior to randomization. The blocks were defined using community characteristics, such as 1) the degree of marginalization (low or medium) 2) the population size and 3) geographic proximity (to avoid large differences in altitude, climate, etc.). Within each block study group, communities were randomly assigned to receive one of the supplements: FF supplied by *PROSPERA*, a micronutrient syrup (syrup) or a micronutrient powder (MNP). The syrup and MNP were purposefully designed with a micronutrient content identical to that of FF. For this analysis, we restricted analyses to the sample of children whose dietary intake data were available at baseline and after four and ten months of supplementation (Fig. 1).

### Study settings and intervention

Physicians and nurses at local healthcare centers and clinics identified PROSPERA beneficiaries for potential inclusion in our study and organized informational meetings regarding our objectives. Prior to initiation, all families in the selected communities had been regularly receiving the PROSPERA FF supplement and health education messages [13]. The nutritional supplements used in this study (FF, MNP and syrup) were delivered directly to the homes of our sample groups six days per week. We did not include a placebo or a no-intervention group because the program, by its rules of operation, includes the distribution of a supplement; therefore, elimination of the supplement was not among the programmatic alternatives to be explored.

The FF, MNP and syrup supplements contained equal amounts of selected micronutrients: elemental iron and zinc, as well as vitamins A, B2, B12, C, E and folic acid (Table 1). FF was formulated with dry whole-milk powder, sugar and maltodextrin; therefore, the children randomized to the FF group were also given 194 kcal of energy, 5.8 g of proteins, 27.9 g of carbohydrates and 6.6 g of lipids per serving.

**Table 1:** Nutritional content per serving for the three nutritional supplements

Nutrient	FF^a^ (44 g)	MNP^b^ (1.0 g)	Syrup (5 mL)
Energy, kcal	194.0	–	–
Protein, g	5.8	–	–
Lipids, g	6.6	–	–
Carbohydrates, g	27.9	–	–
Sodium, mg	24.5	–	–
Iron, mg ^δ^	10.0	10.0	10.0
Zinc, mg	10.0	10.0	10.0
Vitamin A, μg	400.0	400.0	400.0
Vitamin E, mg	6.0	6.0	6.0
Vitamin C, mg	50.0	50.0	50.0
Vitamin B2, mg	0.8	0.8	0.8
Vitamin B12, μg	0.7	0.7	0.7
Folic acid, μg	50.0	50.0	50.0

^a^Fortified Food (FF)

^b^Micronutrient Powder (MNP)

^δ^Gluconate ferrous in FF and Syrup; and fumarate ferrous in MNP

The FF supplement was produced for PROSPERA by Liconsa Corporation, which is located in the Mexican state of Queretaro and was distributed in 264 g bags. Instructions for FF preparation consisted of mixing a daily 44 g dose of dry product with a small amount of water immediately prior to consumption. The MNP supplement was specifically produced for our study and purchased from Ped-Med Ltd. (Toronto, Canada). A recommended daily dose of 1 g was provided in individual packs, and this was to be mixed with a small portion of the child’s food immediately prior to consumption. The syrup supplement was also specifically developed for our project and was donated by Zerboni Laboratories in Mexico City, using a premix donated by DMS Nutritional Products (Mexico City). The syrup was distributed in 60 mL bottles, and a daily dose was 5 mL.

For the first 9 months of the study, the field staff visited each participant daily to distribute the supplement and observe its preparation and consumption. Subsequent visits were scheduled on a weekly basis. During each visit, the mother/caregiver registered the week’s consumption on a self-administered form, which was designed to facilitate the recollection of routine consumption. At each weekly visit, the field staff reviewed and collected the form, gathered any remaining supplements from the previous week, and prepared the child’s doses and form for the following week.

### Data collection

#### Demographic characteristics

Demographic variables were obtained at baseline by means of home interviews with the child’s mother/caregiver. The questionnaire, which was developed for previous studies in Mexico [12], was used to collect information on maternal schooling, occupation, marital status, the family’s socioeconomic status (SES), including housing characteristics, access to water and electricity services, and household possession of durable goods.

#### Dietary assessment

To estimate food consumption, we adapted a previously validated seven-day food frequency questionnaire (FFQ) [19]. The questionnaire was adapted by adding breast milk to the milk group and FF to the cereals group. Although 24-h recall questionnaires applied on nonconsecutive days have been proven to generate a more accurate estimation of individual dietary intake, we used the FFQ as our main objective was to assess patterns of dietary intake and supplement consumption across the groups. Our ability to use more robust dietary assessment methods was also limited by logistical and budgetary restrictions.

The FFQ registered the reported consumption of foods from a list for each child, including the amount consumed and the portion sizes. The questionnaire was administered by trained personnel, with standardized food units and measures, during in-person interviews with the child’s mother/caregiver. The sessions were held at baseline and at follow-up after four and ten months. The FFQ included the 107 most frequently consumed foods according to sample age group in the study population and classified them into 15 groups, with an option to register additional foods not included in the list. The respondent was asked about the number of days and the number of times per day the child consumed each food item and about the amount consumed per serving. These amounts were recorded in different measurement units: ounce, teaspoon, tablespoon, serving tablespoon, drop, gram or milliliter, piece, cup, slice, and taste. During the initial FFQ interview, the mother/caregiver identified the appropriately sized spoons and measuring cups for portion-size estimation.

The number of grams of each food item consumed and its nutritional content were estimated based on the food composition tables of the *Instituto Nacional de Salud Pública* (INSP, according to its Spanish acronym), Mexico [20]. Outliers for plausible intake were excluded according to age and sex to minimize errors in estimating the grams of food consumed [21–23]. We analyzed energy and protein consumed because of their direct relationships with child growth and development. We analyzed iron, zinc and folic acid intake since serum deficiencies of these nutrients have been identified as serious public health concerns in Mexican children [24, 25].

Regarding breast milk, the mothers were asked to report only their breastfeeding status (Yes or No) because we did not attempt to quantify breast milk intake.

### Supplement consumption

Compliance with supplement consumption recommendations was estimated by quarters (25%, 50%, 75%, and 100%) based on the reports provided by the field staff during home visits. For the FF and MNP supplements, the amounts of powder remaining in the individual portions of prepared food were evaluated (0% = all or almost all, 25% = more than half, 50% = approximately half, 75% = less than half, and 100% = nothing or almost nothing). For syrup, the approximate quantity of quarters consumed for portions of 5 mL of syrup was registered. During the first nine months, field staff performed daily visits and registered consumption compliance. Thereafter, field staff performed weekly visits, and forms were completed to cover the whole week. The information was collected weekly. We calculated supplement consumption and compliance during the week before the date of the dietary interviews.

### Statistical analysis

Descriptive statistics were obtained to assess the balance between the study groups in terms of the general characteristics and nutritional status of participants. Means and standard deviations were calculated for quantitative variables, and percentages were calculated for categorical variables. All standard errors were adjusted for data dependencies at the community level using the Taylor Series linearization method [26]. Means were compared through analysis of pairwise differences between study groups using a t-test.

Dietary intake was assessed using median regression with adjusted standard errors for data dependencies at the community level [27]. The model included variables such as study stage, study group, and the interaction between these two parameters. Adjusted medians for each stage (baseline, four-month follow-up, and ten-month follow-up) and study group were obtained from the model equations.

Additional analyses were performed to adjust for variables with unbalanced means between study groups. All interactions of these variables with study stage and study group were included in the model. Adjusted medians were obtained by holding unbalanced covariates at their mean values. All statistical analyses were performed using Stata v.12.0 (Stata Corp. 2011, Stata Statistical Software: Release 12. College Station, TX: StataCorp LP).

## Results

Of the 988 eligible children who completed the supplementation trial, 244 were in the FF group, 326 were in the MNP group, and 296 were in the syrup group. Those who did not provide full dietary data for the three time points (baseline, four months and ten months) were excluded from the analysis, leaving 199 in the FF group, 254 in the MNP group and 238 in the syrup group (Fig. 1). There were no statistically significant differences among those who were included and not included in the analysis for selected characteristics (anthropometric measures, demographic characteristics and SES) (Table 2). No differences in age, sex or anthropometric indicators were observed among the study groups included in this analysis. However, the children in the syrup group began supplementation approximately one month after those in the FF and MNP groups (Table 2), and there was a significant difference in average SES between the FF and syrup groups (*p* < 0.05).

**Table 2:** Baseline characteristics of children included and excluded in this analyses

Characteristics	Included				Excluded *n* = 297
	FF *n* = 199	Syrup *n* = 238	MNP *n* = 254	Total *n* = 691	
Infants					
Age, months	7.9 ± 2.5	8.2 ± 2.7	8.0 ± 2.5	8.0 ± 2.6	8.1 ± 2.5
Sex, % male	46.23	50.84	49.61	49.1	46.8
Weight, kg	7.9 ± 1.1	7.9 ± 1.2	7.9 ± 1.2	7.9 ± 1.2	7.9 ± 1.3
Length, cm	66.9 ± 4.1	67.3 ± 4.3	67.0 ± 4.1	67.1 ± 4.2	67.2 ± 4.6
Length/age, Z	− 1.0 ± 1.0	− 1.1 ± 1.1	− 1.1 ± 1.0	− 1.0 ± 1.1	−1.1 ± 1.1
Length/age, %	15.74	18.07	18.65	17.6	18.5
Starting age of supplementation, months	8.3 ± 2.6	9.1^a,c^ ± 2.8	8.4 ± 2.5	8.6 ± 2.6	–
Mother					
Socioeconomic level	0.2 ± 1.0	− 0.2^b^ ± 1.0	0.0 ± 1.0	0.0 ± 1.0	− 0.1 ± 1.0
Educational level of the mother, %					
None	12.6	10.5	12.2	11.7	7.7
Basic	41.2	45.8	52.4	46.9	50.5
High school	37.7	36.6	26.8	33.3	32.0
Secondary school or more	8.5	7.1	8.7	8.1	9.7

Fortified food (FF), Micronutrient powder (MNP), and Syrup (Syrup)

Mean ± standard deviation or percentages, are presented

^a^*p* < 0.05 FF vs MNP

_b_ *p* < 0.05 FF vs Syrup

^c^*p* < 0.05 Syrup vs MNP

No statistically significant differences were found between children who were the included and not included in the study

### Dietary intake

At baseline, there were no significant differences between the study groups.

**Fig. 2:**
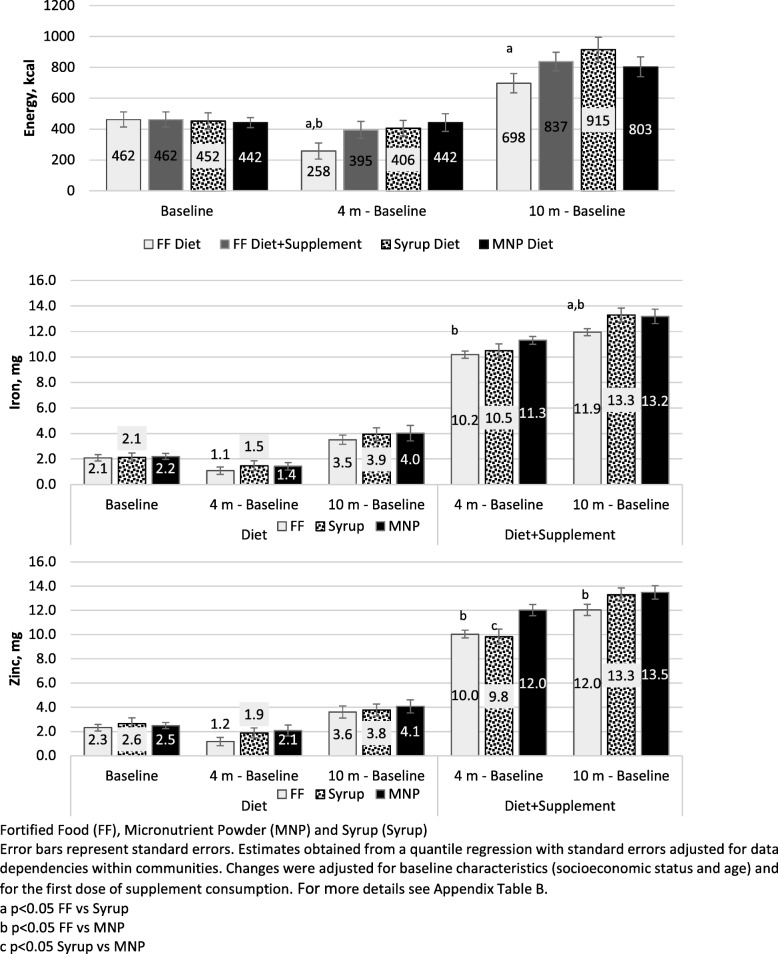
Covariate-adjusted median intake of energy, iron and zinc from diet and diet+supplement at baseline and changes from baseline to follow-up stages by study group

After four months of supplementation, the children in the FF group had smaller increases in median dietary energy (183.7 kcal, CI95%: 59.9, 307.5) and dietary protein (6.6 g, CI95%: 2.6, 10.6) intake from their home diet than those in the syrup group (*p* < 0.05) (Table 3). This energy difference remained significant after adjusting for group differences at baseline (Fig. 2). With regard to covariate-adjusted median changes from baseline to follow-up at 10 months, the children in the FF group had a smaller median increase in dietary energy intake than those in the syrup group (698 vs 915 kcal), with a difference of 217.9 kcal (CI95%: 20.4, 415.4).

**Table 3:** Median intake of energy, protein, iron, zinc and folic acid, from diet at baseline, and changes from baseline to follow-up stages by supplementation group

Stage study	FF	Syrup	MNP	Syrup vs FF	MNP vs FF	MNP vs Syrup
	Diet					
Energy (kcal)						
Baseline	372.7 (258.8, 486.7)	463.1 (363.4, 562.8)	438.9 (333.1, 544.6)	90.4 (−61.0, 241.8)	66.1 (−89.3, 221.6)	− 24.2 (− 169.5, 121.1)
4 m - Baseline	262.4 (179.2, 345.7)	446.1 (354.5, 537.8)	352.2 (253.7, 450.7)	**183.7 (59.9, 307.5)**	89.8 (− 39.2, 218.8)	− 93.9 (− 228.5, 40.6)
10 m - Baseline	784.5 (683.1, 885.9)	955.4 (800.7, 1110.2)	831.9 (703.1, 960.6)	171.0 (− 14.1, 356.0)	47.4 (− 116.5, 211.3)	− 123.6 (− 324.9, 77.7)
Protein (g)						
Baseline	10.1 (7.0, 13.2)	11.8 (8.7, 14.9)	12.8 (9.8, 15.8)	1.7 (−2.7, 6.1)	2.7 (−1.7, 7.1)	1.0 (− 3.4, 5.4)
4 m - Baseline	9.7 (6.4, 13.1)	16.3 (14.1, 18.6)	13.9 (11.3, 16.6)	**6.6 (2.6, 10.6)**	4.2 (− 0.1, 8.5)	−2.4 (− 5.9, 1.1)
10 m - Baseline	28.8 (25.5, 32.1)	32.5 (26.8, 38.2)	28.9 (24.9, 32.9)	3.7 (−2.9, 10.3)	0.1 (−5.1, 5.3)	−3.6 (− 10.6, 3.4)
Iron (mg)						
Baseline	1.9 (1.0, 2.7)	2.0 (1.5, 2.5)	2.2 (1.6, 2.9)	0.2 (−0.8, 1.1)	0.4 (− 0.7, 1.5)	0.2 (− 0.6, 1.1)
4 m - Baseline	1.1 (0.6, 1.6)	1.9 (1.3, 2.6)	1.3 (0.6, 2.1)	0.8 (−0.0, 1.6)	0.2 (−0.7, 1.1)	− 0.6 (− 1.6, 0.4)
10 m - Baseline	4.0 (3.5, 4.6)	4.6 (3.5, 5.8)	4.0 (2.8, 5.2)	0.6 (−0.7, 1.9)	0.0 (− 1.3, 1.3)	−0.6 (−2.3, 1.0)
Zinc (mg)						
Baseline	2.2 (1.5, 2.9)	2.5 (1.8, 3.2)	2.4 (1.8, 3.0)	0.3 (−0.7, 1.3)	0.2 (−0.8, 1.1)	− 0.1 (−1.1, 0.8)
4 m - Baseline	1.1 (0.6, 1.7)	2.2 (1.7, 2.8)	2.1 (1.5, 2.7)	**1.1 (0.4, 1.9)**	**1.0 (0.2, 1.8)**	−0.1 (− 0.9, 0.7)
10 m - Baseline	3.8 (3.1, 4.6)	4.3 (3.0, 5.5)	4.2 (3.2, 5.1)	0.4 (−1.0, 1.9)	0.3 (−0.8, 1.5)	−0.1 (− 1.6, 1.5)
Folic acid (μg)						
Baseline	26.0 (13.4, 38.7)	27.7 (17.4, 38.1)	30.4 (22.8, 38.0)	1.7 (−14.6, 18.0)	4.4 (− 10.4, 19.2)	2.7 (− 10.1, 15.5)
4 m - Baseline	25.3 (13.1, 37.4)	36.1 (17.6, 54.7)	17.7 (7.6, 27.8)	10.9 (− 11.3, 33.1)	− 7.6 (− 23.4, 8.2)	− 18.5 (− 39.6, 2.7)
10 m - Baseline	55.4 (38.6, 72.1)	71.3 (50.1, 92.4)	50.1 (37.3, 63.0)	15.9 (− 11.1, 42.8)	−5.2 (−26.4, 15.9)	− 21.1 (−45.9, 3.6)

Fortified food (FF), Micronutrient powder (MNP), and Syrup (Syrup)

Obtained from a quantile regression with standard errors adjusted for data dependencies within communities; 95% confidence intervals are presented in parentheses

In boldface are the significant differences in differences

### Supplement compliance

The mean supplement compliance during the week before a dietary interview was 83.6% (CI95%: 76.9, 90.4) for the FF group, 84.4% (CI95%: 74.7, 94.1) for the syrup group, and 97.9% (CI95%: 96.5, 99.2) for the MNP group after 4 months of follow-up, and it was 83.7% (CI95%: 79.5, 88.0) for the FF group, 94.3% (CI95%: 91.5, 97.2) for the syrup group, and 93.0% (CI95%: 89.9, 96.1) for the MNP group after 10 months of follow-up.

### Dietary intake with nutrient intake from supplements added to consumption

There were no significant differences between the groups at baseline. At four months of follow-up, the median increase in zinc consumption by the FF group was 2.0 mg (CI95%: 1.0, 2.9) lower than the median increase for the MNP group (*p* < 0.05) (Table 4). After adjusting for SES and supplement initiation age (Fig. 2), the median increase in iron intake after four months of follow-up was 1.1 mg (CI95%: 0.3, 1.9) lower in the FF group than in the MNP group. The median increase in zinc intake was also smaller for the FF group than for the MNP group, with a difference of 2.0 mg (CI95%: 0.9, 3.1). The same tendency for changes in median iron and zinc intake were observed after 10 months of follow-up.

**Table 4:** Median intake of energy, protein, iron, zinc and folic acid; from diet+supplement at baseline, and changes from baseline, to follow-up stages by supplementation group

Stage study	FF	Syrup	MNP	Syrup vs FF	MNP vs FF	MNP vs Syrup
	Diet + supplement					
Energy (kcal)						
Baseline	372.7 (257.2, 488.3)	463.1 (364.1, 562.1)	438.9 (333.6, 544.2)	90.4 (− 61.8, 242.5)	66.1 (− 90.2, 222.5)	− 24.2 (− 168.7, 120.3)
4 m - Baseline	426.6 (325.0, 528.1)	446.1 (353.9, 538.4)	352.2 (253.4, 451.1)	19.6 (− 117.6, 156.8)	− 74.3 (− 216.0, 67.4)	−93.9 (− 229.1, 41.3)
10 m - Baseline	956.9 (848.6, 1065.1)	955.4 (803.4, 1107.4)	831.9 (701.4, 962.4)	−1.4 (− 188.0, 185.2)	−125.0 (− 294.5, 44.6)	−123.6 (− 323.9, 76.8)
Protein (g)						
Baseline	10.1 (7.0, 13.2)	11.8 (8.6, 14.9)	12.8 (9.7, 15.9)	1.7 (−2.7, 6.1)	2.7 (−1.6, 7.0)	1.0 (− 3.4, 5.4)
4 m - Baseline	14.8 (11.5, 18.2)	16.3 (14.1, 18.6)	13.9 (11.2, 16.6)	1.5 (−2.5, 5.5)	− 0.9 (− 5.2, 3.4)	−2.4 (− 5.9, 1.1)
10 m - Baseline	33.5 (29.8, 37.2)	32.5 (26.9, 38.1)	28.9 (24.9, 32.8)	− 1.0 (− 7.7, 5.7)	−4.6 (− 10.1, 0.8)	−3.6 (− 10.4, 3.2)
Iron (mg)						
Baseline	1.9 (1.1, 2.6)	2.0 (1.5, 2.6)	2.2 (1.6, 2.9)	0.2 (− 0.8, 1.1)	0.4 (− 0.6, 1.4)	0.2 (− 0.6, 1.1)
4 m - Baseline	10.4 (9.7, 11.1)	10.7 (9.4, 12.0)	11.1 (10.3, 11.9)	0.3 (− 1.2, 1.7)	0.7 (−0.4, 1.7)	0.4 (− 1.1, 1.9)
10 m - Baseline	12.6 (12.0, 13.1)	13.7 (12.4, 15.0)	13.2 (12.2, 14.3)	1.2 (−0.3, 2.6)	0.7 (−0.5, 1.9)	− 0.5 (− 2.2, 1.2)
Zinc (mg)						
Baseline	2.2 (1.5, 2.9)	2.5 (1.8, 3.3)	2.4 (1.8, 3.0)	0.3 (−0.7, 1.3)	0.2 (−0.8, 1.1)	− 0.1 (−1.1, 0.8)
4 m - Baseline	10.0 (9.3, 10.7)	10.1 (8.9, 11.4)	12.0 (11.4, 12.6)	0.1 (−1.3, 1.6)	**2.0 (1.0, 2.9)**	**1.8 (0.4, 3.3)**
10 m - Baseline	12.6 (11.8, 13.3)	13.6 (12.7, 14.5)	13.5 (12.5, 14.6)	1.0 (− 0.2, 2.2)	1.0 (− 0.3, 2.3)	− 0.0 (− 1.4, 1.4)
Folic acid (μg)						
Baseline	26.0 (13.6, 38.5)	27.7 (16.9, 38.6)	30.4 (22.9, 37.9)	1.7 (−14.8, 18.2)	4.4 (− 10.1, 19.0)	2.7 (− 10.5, 15.9)
4 m - Baseline	62.8 (53.4, 72.1)	79.0 (62.0, 96.1)	65.6 (55.0, 76.2)	16.3 (−3.2, 35.7)	2.8 (− 11.2, 16.9)	− 13.4 (− 33.5, 6.6)
10 m - Baseline	98.8 (83.4, 114.3)	120.1 (92.0, 148.2)	95.2 (82.6, 107.8)	21.3 (− 10.8, 53.3)	−3.6 (− 23.6, 16.3)	− 24.9 (− 55.7, 5.9)

Fortified food (FF), Micronutrient powder (MNP), and Syrup (Syrup)

Obtained from a quantile regression with standard errors adjusted for data dependencies within communities, 95% confidence intervals are presented in parentheses

In boldface are the significant differences in differences

## Discussion

The present study explored median differences in dietary intake of key nutrients by children 6 to 12 months old living in urban areas of Mexico and receiving one of three nutritional supplements, with follow-up after 4 and 10 months. This analysis forms part of a research trial designed to inform improvements to the nutrition component of the *PROSPERA* program. Our findings showed that those receiving FF (with energy and protein supplements provided by the program) consumed less energy, protein and micronutrients from their home diet than those receiving MNP or syrup.

Our study indicates that consumption of FF likely led to displacement of some home foods, resulting in no net contribution to dietary energy and protein intake. Although the supplements themselves were developed with equal micronutrient contents and all three groups showed compliance higher than 80%, the FF group showed lower compliance than the other groups. This observation is likely due to differences in adherence, particularly with regard to the proportion of each dose that was regularly consumed. If adherence in the FF group had been greater, we would expect a greater displacement of the home diet. This finding is consistent with those of several other published studies [28–32].

In the context of urban Mexico, it is likely that energy and protein are not limiting factors in the diets of children. In this setting, the displacement of home foods is desirable to avoid any excess increase in dietary energy. All three supplements provided important contributions to the total intake of several micronutrients that are known to be deficient in the diets of children who are beneficiaries of PROSPERA [25, 33, 34].

Iron supplementation may be indicated for children and women to reduce the occurrence of anemia due to a lack of iron, even in the presence of some known limitations, such as side effects affecting compliance and distribution problems [35]. Providing MNP increases availability and enhances the intake of nutritional supplements. However, granting access to this supplement in the actual setting of program operation is crucial.

A large-scale pilot study was performed in Vietnam, with the purpose of promoting locally produced MNP distribution and making MNP available through sales from the public healthcare system, and the study included training about adequate practices for feeding infants and young children. This type of intervention demonstrated a promising and innovative model for promoting the acquisition and intake of MNP, with the aim of improving nutritional status in a population of children < 5 years old [36]. Other authors demonstrated that children invariably have a more positive response to MNP than to syrup [37, 38]. In a study in Bangladesh in which an MNP intervention was assessed, 60% of mothers stated that they “liked it a lot”, 30% “liked it” and the remaining 10% “somewhat liked it”, particularly because it was easy to prepare, acceptable and could be added to complementary foods [39].

Similarly, with the purpose of increasing MNP compliance and intake, strategies for supplementation programs must be further researched. In this context, a study was performed in Nepal that documented predictors for obtaining high compliance with MNP intake. These predictors included perceived positive effects on health and nutrition in children < 24 months old among caregivers after MNP intake, as well as having specific materials for promoting the adequate intake of supplements. Such predictors should be considered when MNP supplementation programs are designed and applied with the purpose of increasing supplement intake adherence [40]. The use of MNP has been a novel approach for large-scale public healthcare interventions, with percentages of the prescribed MNP packets consumed during the intervention period as high as 98% [41, 42]. Comparatively, a study demonstrated that the dietary energetic density of babies consuming FF was less than that of babies not consuming FF (1.2 kcal/g vs 1.5 kcal/g). These findings can be explained by the fact that the caregivers may not have prepared the supplement according to the recommendations and may have diluted the papilla in an effort to make the supplement last longer [43]. This observation reveals that when MNP is used, there is greater adherence; and among its advantages, the single daily dose of MNP can be added to food that children can eat at any time; thus, greater supplement intake can be achieved.

There are few studies on the effectiveness of food fortification programs [44]. Supplement acceptance, the vehicle used (e.g., papilla preparations) and ease of preparation are key aspects to consider to ensure adoption of a supplement by the target population [45].

It has been documented that protein requirements among children aged 6–23 months are already covered by normal dietary intake, while requirements for energy and other nutrients (e.g., iron, zinc, and calcium) are not met without supplementation [46–48]. Therefore, accurate and effective strategies that contribute to increasing the intake of several nutrients to adequate levels through supplementation are needed. Teaching mothers how to improve feeding with natural food that is readily available in their places of residence has shown important results. However, some key nutrients fail to be covered in this manner, which can have detrimental effects on the weight and height of the child [47, 49, 50]. Therefore, the use of specific micronutrient supplements to cover infants’ daily requirements is essential. In the case of anemia due to a lack of iron, for example, supplements covering iron requirements must be given to children.

According to the sustainable development objectives from the World Health Organization, maternal health is one of the main objectives for supporting healthy lives and promoting lifelong wellbeing. Suggested strategies to prevent maternal-infant malnutrition and nutritional deficiencies during the first thousand days of life (pregnancy and the first two years of life) include the use of prenatal supplements, the promotion of maternal breastfeeding, adequate weaning, the prevention of diseases (such as diarrhea and flu) and specific nutritional interventions with a focus on communities with no food security [47, 51, 52].

Our study has several limitations. The use of an FFQ instrument to assess dietary intake may have led to over-reporting of energy and nutrient consumption. Therefore, it is possible that the absolute nutrient intake levels may be overestimated for our sample. However, the ability to compare dietary intake patterns across the three supplementation groups is not compromised by this general estimation bias. Large proportions (36%) of children were still breastfed after ten months of supplementation. We have no evidence that this feeding influences comparative analyses across supplementation groups. Finally, given the implementation of the study in urban areas and the assessment of dietary intake until only 10 months after the initiation of supplementation, the results cannot be extrapolated to rural areas or to longer supplementation periods. An important strength of the study was the rigorous and continual monitoring of supplement consumption at the household level by highly trained field staff.

## Conclusion

Our study among *PROSPERA* beneficiaries in urban Mexico showed that the consumption of FF led to the displacement of home foods in the diets of children 6 to 24 months of age. These results suggest that diets are generally adequate in terms of food quantity (i.e., energy and protein). All three supplements used in the trial were considered highly acceptable by caregivers [53]. Compliance was higher than 80% in the three supplementation groups, with the MNP and syrup groups showing higher compliance than the FF group during the week before the dietary interview after 10 months of follow-up. Our results suggest that alternative supplements, particularly MNPs or syrups, are more cost effective than FFs in increasing the dietary intake of key micronutrients in the studied population. To determine the implications for PROSPERA, however, these results must also be interpreted in conjunction with the impact of the supplements on nutritional outcomes (results forthcoming).

## Appendix

**Table 5:** Covariate-adjusted* median intake of energy, protein, iron, zinc and folic acid from diet and diet+supplement at baseline and changes from baseline to follow-up stages by supplementation group

Stage study	FF	Syrup	MNP	Syrup vs FF	MNP vs FF	MNP vs Syrup	FF	Syrup	MNP	Syrup vs FF	MNP vs FF	MNP vs Syrup
	Diet						Diet + supplement					
Energy (kcal)												
Baseline	462.0 (365.6, 558.4)	451.9 (348.0, 555.7)	442.0 (376.6, 507.4)	−10.1 (− 151.8, 131.6)	− 20.0 (− 136.5, 96.5)	−9.9 (− 132.6, 112.9)	462.0 (365.9, 558.0)	451.9 (348.4, 555.4)	442.0 (376.8, 507.2)	−10.1 (− 151.3, 131.1)	− 20.0 (− 136.1, 96.1)	−9.9 (− 132.2, 112.5)
4 m - Baseline	257.8 (155.9, 359.8)	405.9 (308.2, 503.5)	442.0 (328.9, 555.1)	**148.0** **(6.9, 289.2)**	**184.1** **(31.9, 336.4)**	36.1 (− 113.3, 185.6)	394.9 (287.4, 502.4)	405.9 (308.4, 503.3)	442.0 (328.1, 555.9)	11.0 (− 134.1, 156.1)	47.1 (− 109.5, 203.7)	36.1 (− 113.8, 186.0)
10 m - Baseline	697.6 (574.9, 820.2)	915.5 (760.6, 1070.3)	802.8 (678.0, 927.5)	**217.9** **(20.4, 415.4)**	105.2 (−69.8, 280.2)	− 112.7 (− 311.5, 86.1)	837.3 (718.9, 955.8)	915.5 (761.2, 1069.7)	802.8 (678.4, 927.1)	78.1 (−116.4, 272.6)	− 34.5 (− 206.3, 137.2)	− 112.7 (− 310.8, 85.5)
Protein (g)												
Baseline	13.1 (10.0, 16.3)	14.2 (10.6, 17.8)	14.6 (11.2, 18.1)	1.1 (−3.7, 5.8)	1.5 (− 3.2, 6.2)	0.4 (− 4.5, 5.4)	13.1 (10.1, 16.2)	12.8 (0.3, 25.2)	11.8 (2.1, 21.5)	−0.4 (− 12.0, 11.2)	−1.3 (−9.9, 7.3)	−0.9 (− 12.0, 10.2)
4 m - Baseline	8.0 (2.7, 13.4)	13.0 (9.2, 16.8)	14.0 (10.1, 17.9)	5.0 (−1.6, 11.5)	6.0 (−0.6, 12.6)	1.0 (− 4.4, 6.5)	21.3 (9.9, 32.7)	22.1 (4.7, 39.5)	15.0 (6.5, 23.6)	0.8 (−20.0, 21.6)	−6.3 (− 20.5, 8.0)	−7.1 (− 26.5, 12.3)
10 m - Baseline	25.3 (18.2, 32.4)	29.8 (24.8, 34.8)	26.9 (22.4, 31.4)	4.5 (−4.1, 13.2)	1.6 (− 6.8, 10.0)	− 2.9 (−9.6, 3.8)	51.7 (25.1, 78.4)	46.8 (26.7, 67.0)	49.6 (37.1, 62.0)	− 4.9 (− 38.3, 28.5)	− 2.2 (− 31.6, 27.2)	2.7 (− 21.0, 26.4)
Iron (mg)												
Baseline	2.1 (1.6, 2.6)	2.1 (1.5, 2.8)	2.2 (1.7, 2.7)	0.0 (−0.8, 0.8)	0.1 (− 0.6, 0.8)	0.1 (− 0.7, 0.9)	2.1 (1.6, 2.6)	2.1 (1.5, 2.8)	2.2 (1.8, 2.6)	0.0 (−0.8, 0.9)	0.1 (− 0.5, 0.8)	0.1 (− 0.7, 0.9)
4 m - Baseline	1.1 (0.5, 1.7)	1.5 (0.7, 2.2)	1.4 (0.9, 2.0)	0.4 (−0.6, 1.3)	0.3 (−0.5, 1.1)	− 0.0 (−1.0, 0.9)	10.2 (9.6, 10.7)	10.5 (9.5, 11.5)	11.3 (10.7, 11.9)	0.3 (−0.9, 1.5)	**1.1** **(0.3, 1.9)**	0.8 (−0.4, 2.0)
10 m - Baseline	3.5 (2.8, 4.2)	3.9 (2.9, 4.9)	4.0 (2.8, 5.2)	0.4 (−0.8, 1.7)	0.5 (−0.9, 1.9)	0.1 (−1.5, 1.6)	11.9 (11.4, 12.5)	13.3 (12.2, 14.4)	13.2 (12.1, 14.3)	**1.4** **(0.1, 2.6)**	**1.2** **(0.0, 2.5)**	−0.1 (−1.7, 1.4)
Zinc (mg)												
Baseline	2.3 (1.8, 2.9)	2.6 (1.7, 3.6)	2.5 (2.0, 3.0)	0.3 (−0.8, 1.4)	0.2 (−0.6, 0.9)	− 0.2 (−1.2, 0.9)	2.3 (1.8, 2.9)	2.6 (1.7, 3.6)	2.5 (2.0, 3.0)	0.3 (−0.8, 1.5)	0.2 (− 0.6, 0.9)	−0.2 (−1.3, 0.9)
4 m - Baseline	1.2 (0.5, 1.8)	1.9 (1.1, 2.7)	2.1 (1.2, 3.0)	0.7 (−0.3, 1.7)	0.9 (−0.2, 2.0)	0.2 (−1.0, 1.4)	10.0 (9.4, 10.7)	9.8 (8.6, 11.1)	12.0 (11.1, 12.9)	−0.2 (−1.6, 1.2)	**2.0** **(0.9, 3.1)**	**2.2** **(0.7, 3.7)**
10 m - Baseline	3.6 (2.6, 4.6)	3.8 (2.8, 4.7)	4.1 (3.0, 5.1)	0.2 (−1.2, 1.5)	0.5 (−1.0, 1.9)	0.3 (− 1.1, 1.7)	12.0 (11.1, 13.0)	13.3 (12.2, 14.4)	13.5 (12.4, 14.6)	1.3 (−0.2, 2.7)	**1.4** **(0.0, 2.9)**	0.2 (−1.3, 1.7)
Folic acid (μg)												
Baseline	29.6 (22.4, 36.9)	28.3 (19.9, 36.7)	33.5 (25.1, 41.9)	−1.3 (−12.4, 9.8)	3.9 (−7.2, 15.0)	5.2 (−6.6, 17.1)	29.6 (22.3, 37.0)	28.3 (19.7, 36.9)	33.5 (25.3, 41.8)	−1.3 (− 12.6, 10.0)	3.9 (−7.1, 14.9)	5.2 (− 6.7, 17.1)
4 m - Baseline	15.0 (5.0, 25.0)	29.9 (17.7, 42.2)	12.2 (0.4, 24.1)	14.9 (−0.9, 30.7)	−2.8 (−18.3, 12.7)	**− 17.7** **(−34.7, − 0.7)**	56.5 (45.3, 67.8)	70.3 (59.4, 81.2)	60.1 (47.9, 72.2)	13.8 (− 1.9, 29.4)	3.6 (− 13.0, 20.1)	−10.2 (− 26.5, 6.1)
10 m - Baseline	54.6 (44.6, 64.6)	70.7 (49.9, 91.5)	44.6 (29.5, 59.8)	16.1 (−7.0, 39.2)	−10.0 (−28.1, 8.1)	**− 26.1** **(− 51.8, − 0.4)**	99.2 (86.7, 111.7)	116.8 (96.9, 136.8)	91.4 (75.1, 107.8)	17.6 (− 5.9, 41.2)	− 7.7 (− 28.3, 12.9)	−25.4 (− 51.2, 0.4)

Fortified food (FF), Micronutrient powder (MNP), and Syrup (Syrup)

Obtained from a quantile regression with age at the onset of supplementation and socioeconomic level as adjustment covariates and standard errors adjusted for data dependencies within communities. *Age at the onset of supplementation and socioeconomic status level

In boldface are the significant differences in differences

## Abbreviations

ENSANUT: (according to its Spanish acronym): National Health and Nutrition Survey
FF: Fortified food
FFQ: Food frequency questionnaire
INSP: (according to its Spanish acronym): National Institute of Public Health, Mexico
Kcals: Kilocalories
MNP: Micronutrient powder
PROGRESA: Education, Health and Alimentation Program
SES: Socioeconomic status

## References

[1] Micronutrient Initiative, Flour fortification initiative, USAID, GAIN, WHO, the World Bank, UNICEF. Investing in the future: a united call to action on vitamin and mineral deficiencies: global report 2009. Ottawa: The Micronutrient Initiative; 2009.

[2] UNICEF. Investing in the future. A united call to action on vitamin and mineral deficiencies. 2009. http://www.unitedcalltoaction.org/documents/Investing_in_the_future.pdf of subordinate document. Accesed 14 Oct 2016.

[3] Adu-Afarwuah S, Lartey A, Brown K (2007). Randomized comparison of 3 types of micronutrient supplements for home fortification of complementary foods in Ghana: effects on growth and motor development. Am J Clin Nutr.

[4] Stein A, Barnhart H, Hickey M, Ramakrishnan U (2003). Prospective study of protein-energy supplementation early in life and of growth in the subsequent generation in Guatemala. Am J Clin Nutr.

[5] Ramakrishnan U, Neufeld L, Flores R, Rivera J, Martorell R (2009). Multiple micronutrient supplementation during early childhood increases child size at 2 y of age only among high compliers. Am J Clin Nutr.

[6] Brown K, Sánchez-Griñan M, Pérez F (1995). Effects of dietary energy density and feeding frequency on total daily energy intakes of recovering malnourished children. Am J Clin Nutr.

[7] Ramakrishnan U, Nguyen P, Martorell R (2009). Effects of micronutrients on growth of children under 5 y of age: meta-analyses of single and multiple nutrient interventions. Am J Clin Nutr.

[8] Galloway R, McGuire J (1994). Determinants of compliance with iron supplementation: supplies, side effects, or psychology?. Soc Sci Med.

[9] Ramakrishnan U, Goldenberg T, Allen L (2011). Do multiple micronutrient interventions improve child health, growth, and development?. J Nutr.

[10] Shamah-Levy T, Villalpando S, Jáuregui A, Rivera JA (2012). Overview of the nutritional status of selected micronutrients in Mexican children in 2006. Salud Publica Mex.

[11] Gutiérrez JP, Rivera-Dommarco J, Shamah-Levy T, Villalpando-Hernández S, Franco A, Cuevas-Nasu L, Romero-Martínez M, Hernández-Ávila M. Encuesta Nacional de Salud y Nutrición 2012. Resultados Nacionales. Cuernavaca: Instituto Nacional de Salud Pública; 2012 https://ensanut.insp.mx/informes/ENSANUT2012ResultadosNacionales.pdf. Accessed 19 Oct 2016.

[12] Neufeld L, Sotres-Alvarez D, Flores-López L, Tolentino-Mayo L, Jiménez-Ruiz J, Rivera-Dommarco J (2005). Estudio sobre el consumo de los suplementos Nutrisano y Nutrivida en niños y mujeres de zonas urbanas beneficiarios de Oportunidades. Evaluación externa de impacto del Programa Oportunidades 2004.

[13] Secretaria de Desarrollo Social (SEDESOL). Reglas de Operación del Programa de Desarrollo Humano Oportunidades, para el ejercicio fiscal 2012. Diario Oficial, tercera sección 2012. Accessed 14 Oct 2016.

[14] Rivera JA, Sotres-Alvarez D, Habicht JP, Shamah T, Villalpando S (2004). Impact of the Mexican program for education, health and nutrition (Progresa) on rates of growth and anemia in infants and young children: a randomized effectiveness study. JAMA.

[15] Neufeld LM, Steta C, Rivera J, Valle AM, Grados R, Uriega S, López VH (2011). Evaluation for program decision making: a case study of the Oportunidades program in Mexico. J Nutr.

[16] Leroy J, Vermandere H, Neufeld L, Bertozzi S (2008). Improving enrollment and utilization of the Oportunidades program in Mexico could increase its effectiveness. J Nutr.

[17] García-Guerra A, Neufeld L, Domínguez-Islas CP, García Feregrino R, HernándezCabrera A (2008). Effect of three supplements with identical micronutrient content on anemia in Mexican children. FASEB J.

[18] García-Guerra A, Rivera-Dommarco J, Neufeld LM, Domínguez-Islas CP (2009). Effect of three supplements with equal micronutrient content on serum zinc concentrations in Mexican children. FASEB J.

[19] Hernández AM, Romieu I, Parra S, Hernández JA, Madrigal H, Willet W (1998). Validity and reproducibility of a food frequency questionnaire to assess dietary intake of women living in Mexico City. Salud Publica Mex.

[20] Safdie M, Barquera S, Porcayo M, et al., Inventors. Bases de datos del valor nutritivo de los alimentos. Compilación del Instituto Nacional de Salud Pública 2004.

[21] Rodrıguez-Ramırez S, Mundo-Rosas V, Jimenez-Aguilar A, Shamah-Levy T (2009). Methodology for the analysis of dietary data from the Mexican National Health and nutrition survey 2006. Salud Publica de Mexico.

[22] Food and Nutrition Board IOM (2001). Dietary Refrence Intake for Vitamin A, Vitamina K, Arsenic, Boron, Chromium, Copper, Iodine, Iron, Manganese, Milybdenum, Nickel, Silicon, Vanadium and Zinc.

[23] Food and Nutrition Board IOM (2005). Dietary Refrence Intake for Energy,Carbohydrate, Fiber, Fat, Fatty Acids, Cholesterol, Proteín and Amino Acids.

[24] Morales-Ruán M, Del C, Villalpando S, García-Guerra A, Shamah-Levy T, Robledo-Pérez R, Avila-Arcos MA, Rivera JA (2012). Iron, zinc, copper and magnesium nutritional status in Mexican children aged 1 to 11 years. Salud Publica Mex.

[25] Cuevas-Nasu L, Mundo-Rosas V, Shamah-Levy T, Méndez-Gómez Humaran I, Avila-Arcos MA, Rebollar-Campos Mdel R, Villalpando S (2012). Prevalence of folate and vitamin B12 deficiency in Mexican children aged 1 to 6 years in a population-based survey. Salud Publica Mex.

[26] Lohr SL (2009). Complex surveys in: Lohr SL. Sampling: design and analysis. 2nd ed.

[27] Parente PMDC, Santos Silva JMC (2016). Quantile regression with clustered data. J of Eco Meth.

[28] Islam MM, Khatun M, Peerson JM, Ahmed T, Mollah MA, Dewey KG, Brown KH (2008). Effects of energy density and feeding frequency of complementary foods on total daily energy intakes and consumption of breast milk by healthy breastfed Bangladeshi children. Am J Clin Nutr.

[29] Diana A, Mallard SR, Haszard JJ, Purnamasari DM, Nurulazmi I, Herliani PD (2017). Consumption of fortified infant foods reduces dietary diversity but has a positive effect on subsequent growth in infants from Sumedang district, Indonesia. PLoS ONE.

[30] Campbell RK, Hurley KM, Shamim AA, Shaikh S, Chowdhury ZT, Mehra S, de Pee S, Ahmed T, West KP, Christian P (2016). Effect of complementary food supplementation on breastfeeding and home diet in rural Bangladeshi children. Am J Clin Nutr.

[31] Maleta K, Kuittinen J, Duggan MB, Briend A, Manary M, Wales J, Kulmala T, Ashorn P (2004). Supplementary feeding of underweight, stunted Malawian children with a ready-to-use food. J Pediatr Gastroenterol Nutr.

[32] Thakwalakwa CM, Ashorn P, Phuka JC, Cheung YB, Briend A, Maleta KM (2015). Impact of lipid-based nutrient supplements and corn-soy blend on energy and nutrient intake among moderately underweight 8-18-month-old children participating in a clinical trial. Matern Child Nutr.

[33] Ramírez-Silva I, Rivera J, Leroy J, Neufeld L (2013). The Oportunidades programs fortified food supplement, but not improvements in the home diet, increased the intake of key micronutrients in rural Mexican children aged 12–59 months. J Nutr.

[34] Villalpando S, de la Cruz V, Shamah-Levy T, Rebollar R, Contreras-Manzano A (2015). Nutritional status of iron, vitamin B12, folate, retinol and anemia in children 1 to 11 years old. Results of the Ensanut 2012. Salud Publica Mex.

[35] Mora JO (2002). Iron supplementation: overcoming technical and practical barriers. J Nutr.

[36] Nguyen M, Poonawala A, Leyvraz M, Berger J, Schofield D, Nga TT, Van TK, do TB H, Wieringa FT (2016). A Delivery Model for Home Fortification of Complementary Foods with Micronutrient Powders: Innovation in the Context of Vietnamese Health System Strengthening. Nutrients.

[37] Zlotkin SH, Arthur P, Antwi KY, Yeung G (2001). Treatment of anemia with microencapsulated ferrous fumarate plus ascorbic acid supplied as ‘sprinkles’ to complementary (weaning) foods. Am J Clin Nutr.

[38] Zlotkin SH, Schauer C, Christofides A, Sharieff W, Tondeur MC, et al. Micronutrient Sprinkles to Control Childhood Anaemia. Micronutrient Sprinkles to Control Childhood Anaemia. PLOS Medicine. 2005;2(1). 10.1371/journal.pmed.0020001. 10.1371/journal.pmed.0020001 PMC545194 15696200

[39] Hyder SMZ, Zlotkin SH, Haseen F, Zeng L. Efficacy of daily vs. weekly home fortification of weaning foods with Sprinkles among infants and young children in Dhaka, Bangladesh. In: Workshop proceedings from the National Workshop on Home Fortification of Weaning Food with Sprinkles: A new strategy to control iron deficiency anemia among infants and young children; 2004. Dhaka: Bangladesh rural advancement committee. p 1–24 http://research.brac.net/reports/2004_sprinkles_efficacy_report.pdf. Accessed 21 May 2018.

[40] Mirkovic KR, Perrine CG, Subedi GR, Mebrahtu S, Dahal P, Staatz C, Jefferds ME (2016). Predictors of micronutrient powder intake adherence in a pilot programme in Nepal. Public Health Nutr.

[41] Ciomarten T, Nanu R, Iorgulascu D, Moldovanue F, Popa S, Palicari G, Nestel P (1995). Iron supplement trial in Romania. Proceedings, Iron Interventions for Child Survival.

[42] Nestel P, Alnwick D (1997) Iron/multi-micronutrient supplements for young children. Summary and conclusions of consultation. UNICEF, Copenhagen, August 19–20, 1996. Washington, DC: International Life Sciences Institute.

[43] Ip H, Hyder S, Haseen F, Rahman M, Zlotkin S (2009). Improved adherence and anaemia cure rates with flexible administration of micronutrient sprinkles: a new public health approach to anaemia control. Eur J Clin Nutr.

[44] Martorell R, de Romaña DL (2017). Components of successful staple food fortification programs: lessons from Latin America. Food Nutr Bull.

[45] Neufeld LM, Baker S, Garrett GS, Haddad L (2017). Coverage and utilization in food fortification programs: critical and neglected areas of evaluation. J Nutr.

[46] Diana A, Mallard SR, Haszard JJ, Purnamasari DM, Nurulazmi I, Herliani PD (2017). Consumption of fortified infant foods reduces dietary diversity but has a positive effect on subsequent growth in infants from Sumedang district, Indonesia. PLoS ONE.

[47] Dewey KG (2013). The challenge of meeting nutrient needs of infants and young children during the period of complementary feeding: an evolutionary perspective. J Nutr.

[48] Osendarp SJ, Broersen B, van Liere MJ, De-Regil LM, Bahirathan L, Klassen E, Neufeld LM (2016). Complementary feeding diets made of local foods can be optimized, but additional interventions will be needed to meet Iron and zinc requirements in 6- to 23-month-old children in low- and middle-income countries. Food Nutr Bull.

[49] Vossenaar M, Solomons NW (2012). The concept of "critical nutrient density" in complementary feeding: the demands on the "family foods" for the nutrient adequacy of young Guatemalan children with continued breastfeeding. Am J Clin Nutr.

[50] Dewey KG (2016). Reducing stunting by improving maternal, infant and young child nutrition in regions such as South Asia: evidence, challenges and opportunities. Matern Child Nutr..

[51] Bhutta ZA, Ahmed T, Black RE, Cousens S, Dewey K, Giugliani E, Haider BA, Kirkwood B, Morris SS, Sachdev HPS, Shekar M (2008). Maternal and Child Undernutrition Study Group. What works? Interventions for maternal and child undernutrition and survival. Lancet.

[52] Miller S, Abalos E, Chamillard M, Ciapponi A, Colaci D, Comandé D, Diaz V, Geller S, Hanson C, Langer A, Manuelli V, Millar K, Morhason-Bello I, Castro CP, Pileggi VN, Robinson N, Skaer M, Souza JP, Vogel JP, Althabe F (2016). Beyond too little, too late and too much, too soon: a pathway towards evidence-based, respectful maternity care worldwide. Lancet.

[53] Flores L, Théodore F, Bonvecchio A, Blanco I, Neufeld LM (2008). Acceptability of three supplements with identical micronutrient content in Mexican children. FASEB J.

